# Metabolic Evaluation in Patients With Hepatitis C Treated With Direct Antiviral Agents

**DOI:** 10.3389/fmed.2021.631600

**Published:** 2021-05-31

**Authors:** Sergio Estefan, Carlos Eduardo Brandão-Melo, Cintia Marques dos Santos Silva, Danilo Cosme Klein Gomes, Paula Cardoso, Marcia Helena S. Costa

**Affiliations:** Endocrinology and Hepatology Division of Federal University of Rio de Janeiro State, Rio de Janeiro, Brazil

**Keywords:** hepatitis C, metabolic syndrome, hepatic steatois, direct action antiviral agents, hepatic fibrosis

## Abstract

Epidemiological data clearly indicate a link between hepatitis C virus (HCV) and altered glucose homeostasis.

**Objective:** To evaluate the response of treatment with direct antiviral agents (DAAs) on metabolic variables of patients with hepatitis C.

**Methods:** Observational, cross-sectional study in a sample of patients with hepatitis C starting therapy with DAAs followed on the hepatology division of Federal University of Rio de Janeiro State. Data were collected in two stages: before the start of therapy and between 12 and 52 weeks after obtaining the sustained virological response.

**Results:** In the baseline assessment of the 97 patients selected, 19.3% were obese, 38.6% were overweight, 50% were hypertensive, 43.8% were pre-diabetic, 12.5% were diabetic, 31.2% were dyslipidemic, and 21.8% had metabolic syndrome. There was an increase in total cholesterol and LDL levels (*p* < 0.001), and a non-significant reduction in blood glucose, glycated hemoglobin, insulin, and HOMA-IR levels after treatment. In the post-treatment, there was a reduction in fibrosis (*p* = 0.016), with a reduction in the levels of GGT, AST, and ALT (all with *p* < 0.001), as well as in the FIB4 and APRI scores (both with *p* < 0.001) and in the degree of fibrosis evaluated by elastography represented in kPa (*p* = 0.006). The blood glucose level was higher in patients with steatosis (*p* = 0.039) after treatment. There was a positive pre-treatment correlation between the degree of fibrosis (kPa) and FIB4 (*r* = 0.319, *p* = 0.004), APRI (*r* = 0.287, *p* = 0.010), and the NAFLD score (*r* = 0.275, *p* = 0.016).

**Conclusion:** Patients with hepatitis C had a high prevalence of metabolic disturbance in the pre-treatment phase, but the therapy did not show beneficial effects, especially on glucose metabolism.

## Introduction

Currently, it is estimated that the prevalence of hepatitis C virus (HCV) is 1.0% of the world population, corresponding to about 71 million active cases (1). In 2016, the 69th World Health Assembly approved the Global Health Sector Strategy to eliminate hepatitis infection by 2030, and WHO introduced global targets for HCV care and management, including a 90% reduction in new cases of this disease, chronic hepatitis, 65% reduction in hepatitis deaths, and treatment of 80% of eligible people with chronic hepatitis C infections (2). The ultimate goal of treating HCV infection is a sustained virological response (SVR). The therapeutic management of HCV has recently shifted from interferon-based therapies to combination regimes of direct-acting antiviral agents (DAAs) interferon-free. DAAs are a new class of drugs directed to non-structural proteins responsible for HCV replication and infection (3). Obtaining an SVR is associated with regression of liver fibrosis, and in patients with cirrhosis, reduced portal hypertension and attenuated risk of developing liver decompensating and chronic hepatitis C (HCC) (4). The introduction of DAAs in recent years has revolutionized the HCV infection treatment landscape. Currently, SVR can be achieved in almost all patients treated with oral regimens combined with minimal side effects (5).

Despite the SVR, some patients remain with clinically significant steatosis and fibrosis. Liver steatosis is commonly found in individuals with chronic HCV, occurring in approximately 50% of liver biopsy samples, with a reported range of 30 to 70%, suggesting that HCV has a direct role in the development of steatosis (6). The increased metabolic risk associated with the presence of hepatic steatosis is commonly found in individuals with chronic hepatitis C and is supported by cross-sectional and longitudinal studies (7).

Epidemiological data clearly indicate a link between HCC and impaired glucose homeostasis. The prevalence of type 2 diabetes mellitus (DM2) and insulin resistance (IR) are higher among people chronically infected with hepatitis C when compared to the general population and other causes of chronic liver disease (8). The presence of concomitant risk factors specific to the host also contributes to the prevalence and degree of metabolic disorders presented; the coexistence of obesity or metabolic syndrome (MS) is an additional risk for the development of glucose homeostasis disorders among patients with chronic hepatitis (9). Although the association of HCV with diabetes mellitus has been confirmed, its underlying mechanism remains unknown. Some of these mechanisms include the direct interaction of HCV proteins with the hepatocyte insulin signaling cascade (6).

If HCV is directly involved in the development of IR and MS in these patients, its clearance should result in a parallel decrease in the risk of the incidence of DM2.

Consequently, successful HCV eradication could improve IR, glycemic control, and clinical outcomes in patients with established DM2 (10).

In this way, better understanding the metabolic profile of patients beginning therapy with DAAs and the impact of treatment on metabolic variables is of paramount importance for the indirect control of liver disorders, having seen its known association with genesis of non-alcoholic fatty liver disease.

## Patients and Methods

Observational, cross-sectional study, included patients, men and women between 18 and 85 years old, selected from a convenience sample of hepatitis C patients followed on the hepatology division of Federal University of Rio de Janeiro State.

People with other viral hepatitis and HCV/HIV co-infected, neoplastic disease, chronic renal failure, HbA1c > 9.5%, pregnant women, patients without SVR, patients with alcohol intake >40 g/day for men and 20 g/day for women were excluded.

Data were collected in two stages, before the start of therapy and between 12 and 52 weeks, after obtaining the SVR, defined as an undetectable serum level of HCV RNA 12 weeks after the end of the therapy. Eligible patients were treated with DAAs, according to scheme recommended by Brazilian Ministry of Health through the Clinical Protocol and Therapeutic Guidelines for Hepatitis C and Co-infections, for a period of 12 weeks.

Of the 97 selected patients, data regarding physical examination [weight, height, body mass index (BMI), and blood pressure] and data from laboratory tests were collected (HCV/RNA viral load, lipid profile, insulin, liver transaminases, fasting glucose, blood count, total bilirubins and fractions, prothrombin time, total protein and albumin, gamma glutamyl transferase, alkaline phosphatase, genotyping HCV, and glycated hemoglobin), as well as assessment of liver fibrosis by indirect methods (NAFLD Score, APRI, FIB-4, Transient Liver Elastography). During the evaluation interval, there was no specific intervention in the therapeutic regimen of patients' underlying diseases, as well as no medication class was a limiting factor for entering the study.

The characterization of metabolic variables was proposed according to the following definitions: Pre-diabetes, a clinical condition of increased risk for diabetes and encompassing the conditions previously called “altered fasting glucose” and “impaired glucose tolerance,” based on the last Official Positioning Brazilian Diabetes Society (11) n 01/2019 as fasting blood glucose between 100 and 125 mg/dl or HbA1c between 5.7 and 6.4%; Diabetes, defined in patients with previous diagnosis and used criteria for diabetes proposed by the American Diabetes Association (12): fasting glucose ≥126 mg/dl or HbA1c ≥6.5%. Although the hyperinsulinemic euglycemic clamp is considered the gold standard method for assessing insulin resistance, this is a complex method limited to some *in vivo* metabolism studies today, so HOMA is a substitute for assessing insulin resistance; it was calculated using the HOMA index, described by Matthews et al. (13), for the estimation of insulin resistance, in which HOMA-RI = fasting insulinemia (mU/L) × fasting glycemia (mmol/L)/22.5; Arterial hypertension, according to the latest guideline of the American College of Cardiology/American Heart Association (14) of 2019 on the primary prevention of cardiovascular diseases, was used as the defining criterion, patient being treated or blood pressure >130 × 80 mmHg; Metabolic Syndrome, due to the limitation of anthropometric data, as well as for dyslipidemia, the parameters used were the criteria adopted by the World Health Organization published in 1998 (15), presence of glucose intolerance or diabetes mellitus, associated with at least two more changes between arterial hypertension, dyslipidemia (triglycerides) >150 mg/dl, and/or HDL <35 mg/dl for men and <39 mg/dl for women; and Central obesity, defined by a BMI >30.0 kg/m^2^.

The assessment of hepatic fibrosis was performed by indirect methods: NAFLD Score, APRI, FIB-4, Transient Elastography (TE), based on previous validation studies. For TE, cutoff values used were 7.1 kPa for *F* > or = 2; 9.5 kPa for *F* > or = 3; and 12.5 kPa for *F* = 4, described by Castéra et al. (16). For a sensitivity ≥ 90%, the CAP cutoff points were 215 dB m^−1^ for *S* ≥ 1; 252 dB m^−1^ for *S* ≥ 2; and 296 dB m^−1^ for S ≥ 3, described by de Lédinghen et al. (17).

In fibrosis scores, we used for the NAFLD Score the regression formula described by Angulo et al. (18) in 2007; NAFLD fibrosis score = −1.675 + 0.037 × age (years) + 0.094 × BMI (kg/m^2^) + 1.13 × IFG/diabetes (yes = 1, no = 0) + 0.99 × AST/ALT ratio – 0.013 × platelets (× 109/l) – 0.66 × albumin (g/dl). We applied the low cutoff point (−1.455) to exclude advanced fibrosis and the high cutoff point (0.676) to confirm the presence of advanced fibrosis. For the APRI score, the regression formula described by Wai et al. (19) is calculated as follows: APRI = [AST level (/ULN)/platelet count (10^9^/L)] × 100. We applied the low cutoff point (<0.5) to exclude advanced fibrosis and the high cutoff point (≥1.5) to confirm advanced fibrosis. For the FIB-4 score, a regression formula described by Sterling et al. (20) is used, calculated with the following formula: FIB-4 = [age (years) × AST (UI/L)]/[platelet count (10^9^/L) × ALT (UI/L)] 1/2. We applied the low cutoff point (<1.45) to exclude advanced fibrosis and the high cutoff point (>3.25) to confirm advanced fibrosis.

The study was approved by the Research Ethics Committee of Federal University of Rio de Janeiro State.

### Statistical Analysis

Qualitative variables were represented as absolute and relative frequencies, and quantitative variables were represented as mean ± standard deviation. Quantitative variables were submitted to the Shapiro–Wilk normality test. For comparisons of quantitative variables before and after treatment, the Wilcoxon or Student *t*-tests for paired samples were used. In comparing quantitative variables between individuals with steatosis present and absent, the Wilcoxon Mann–Whitney test was adopted for independent samples. To evaluate steatosis and fibrosis before and after treatment, the binary logistic model was used. The correlation between quantitative variables was performed via Spearman's correlation coefficient, with respective significance test. The analyses were performed using the free program R version 3.5.2 and *p* < 0.05 was considered significant.

## Results

The sample consisted of 97 patients, 51.5% of whom were male. The mean age was 57.9 ± 13.3 years, and the mean BMI was 26.7 ± 4.9 kg/m^2^. The most common genotype was type 1 (87.6%) with subtype 1B (43.3%) being the most prevalent. The mean HCV-RNA value was 2,192,797 ± 4,280,313 IU/ml. In the evaluation of the baseline sample, 19.3% were obese, 38.6% were overweight, and the prevalence of arterial hypertension was observed in 50% of the patients, 43.8% in pre-diabetes, 12.5% in diabetes, 31.2% in dyslipidemia, and 21.8% in metabolic syndrome. The mean value of the NAFLD score was −1.28 ± 1.68 (Table 1).

**Table 1:** Pre-treatment sample.

**Characteristics**	***N***	**Statistics**
Sex	97	
Male		50 (51.5%)
Age	97	57.9 ± 13.3
BMI (kg/m^2^)	88	26.7 ± 4.9
Genotype	97	
1		15 (15.5%)
1A		22 (22.7%)
1B		42 (43.3%)
1A/1B		5 (5.2%)
2B		1 (1%)
3		4 (4.1%)
3A		6 (6.2%)
4		2 (2.1%)
HCV-RNA (UI/ml)	96	2,192,797 ± 4,280,313
Obesity	88	17 (19.3%)
Overweight	88	34 (38.6%)
Hypertension	84	42 (50%)
Pre-diabetes	96	42 (43.8%)
Diabetes	96	12 (12.5%)
Dyslipidemia	93	36 (38.7%)
Metabolic syndrome	78	17 (21.8%)
NAFLD Score	75	−1.28 ± 1.68

*BMI, Body mass index; NAFLD, Non-Alcoholic Fatty Liver Disease*.

In the evaluation of the metabolic profile before and after treatment, there was an increase in the levels of total cholesterol (TC) (*p* < 0.001), LDL (*p* < 0.001), and triglycerides (TGD) (*p* = 0.047) and a non-significant reduction in the levels of glycemia, glycated hemoglobin, insulin, and HOMA-IR. In assessing the degree of liver fibrosis, there was a reduction in the levels of GGT, AST, and ALT (all with *p* < 0.001); a reduction in the FIB4 and APRI scores (both with *p* < 0.001); and the degree of fibrosis was evaluated by elastography represented in kPa (*p* = 0.006; Table 2).

**Table 2:** Evaluation of pre- and post-treatment laboratory data.

**Variable**	**Pre-treatment**	**Post-treatment**	***p*-value**
Glycemia	99.0 ± 21.9	96.4 ± 18.9	0.082^W^
HbA1c	5.50 ± 0.62	5.32 ± 0.98	0.365^W^
Insulin	15.18 ± 12.62	14.46 ± 8.52	0.765^W^
HOMA-IR	3.69 ± 3.28	3.56 ± 2.18	0.331^W^
Total colesterol	162 ± 30.8	188.3 ± 49.6	<0.001^W^
HDL	50.8 ± 15.9	50.5 ± 13.8	0.601^W^
LDL	92.6 ± 26.7	114.9 ± 41.5	<0.001^W^
TGD	94.4 ± 46.8	111.9 ± 62.7	0.047^W^
GGT	72.3 ± 69.5	30.4 ± 26.5	<0.001^W^
AST	56.3 ± 37.1	23.3 ± 7.9	<0.001^W^
ALT	69.9 ± 50.5	22.0 ± 10.3	<0.001^W^
FIBROSES (kPa)	10.93 ± 11.70	8.94 ± 5.36	0.006^W^
FIB4	2.78 ± 3.33	1.66 ± 1.38	<0.001^W^
APRI	1.05 ± 1.25	0.37 ± 0.30	<0.001^W^
CAP	207 ± 44.47	234.7 ± 51.01	0.098^T^

W *Wilcoxon test for paired sample. ^T^Student t-test for paired sample. HbA1c, glycated hemoglobin; HDL, high-density lipoprotein; LDL, low-density lipoprotein; TGD, triglycerides; GGT, gamma glutamyl transferase; AST, aspartate aminotransferase; ALT, alanine aminotransferase; kPa, kilopascals; FIB4, The Fibrosis-4 Index; APRI, AST to Platelet Ratio Index; CAP, controlled attenuation parameter*.

In an analysis including only patients with response at both times, there was no significant change in the distribution of steatosis (*n* = 21) before and after treatment. There was a reduction in individuals with fibrosis (*n* = 26) (*p* = 0.011); more specifically, there was a reduction in advanced fibrosis F3 (*p* = 0.015) and F4 (*p* = 0.014) in the post-treatment (Table 3). The presence of steatosis was not related to blood glucose, insulin, HbA1c, and HOMA-IR levels in the pre-treatment. The levels of glycemia, insulin, HbA1c, and HOMA-IR did not change with treatment among individuals with steatosis at both times. After treatment, the blood glucose level was higher in patients with steatosis (*p* = 0.039) (Table 4).

**Table 3:** Stratification of steatosis and fibrosis levels in patients pre- and post-treatment.

**Variable**	**Pre-treatment**	**Post-treatment**	***p*-value**
Steatosis (*n* = 21)	*n* = 10	*n* = 14	0.215
S0	11 (52.4%)	7 (33.3%)	–
S1	7 (33.3%)	6 (28.6%)	0.686
S2	1 (4.8%)	5 (23.8%)	0.085
S3	2 (9.5%)	3 (14.3%)	0.407
Fibrosis (*n* = 26)	*n* = 22	*n* = 13	0.011
F0–F1	4 (15.4%)	13 (50%)	–
F2	4 (15.4%)	6 (23.1%)	0.370
F3	6 (23.1%)	1 (3.8%)	0.015
F4	12 (46.2%)	6 (23.1%)	0.014

*The p-values refer to the binary logistic model. Only patients with responses in both moments*.

**Table 4:** Relationship between the degree of steatosis and the variables glycemia, insulin, HbA1c, and HOMA-IR pre- and post-treatment.

**Variable**	**Pre-treatment**	**Post-treatment**	***p*-value^**2**^**
**Glycemia**			
With steatosis	88.4 ± 12.0	101.8 ± 28.7	0.587
Without steatosis	94.4 ± 12.1	87.57 ± 8.1	–
*p*-value^1^	0.323	0.039	
**Insulin**			
With steatosis	19.5 ± 12.8	16.6 ± 9.1	0.906
Without steatosis	12.4 ± 6.7	11.0 ± 6.1	–
*p*-value^1^	0.351	0.154	
**HbA1c**			
With steatosis	5.48 ± 0.31	5.34 ± 1.57	0.755
Without steatosis	5.59 ± 0.38	5.4 ± 0.23	–
*p*-value^1^	0.476	0.415	
**HOMA-IR**			
With steatosis	4.07 ± 2.43	4.16 ± 2.08	0.946
Without steatosis	2.90 ± 1.65	2.35 ± 1.35	–
*p*-value^1^	0.424	0.058	

*The p-values^1^ refer to the comparison with and without Steatosis (Wilcoxon Mann–Whitney test for independent samples), and the p-values^2^ refer to the pre- and post-treatment comparison (Wilcoxon test for paired samples). Only patients with responses in both times*.

There was a positive pre-treatment correlation between the degree of fibrosis assessed by TE represented in kPa and FIB4 (*r* = 0.319, *p* = 0.004), APRI (*r* = 0.287, *p* = 0.010), and the NAFLD score (*r* = 0.275, *p* = 0.016).

## Discussion

The baseline demographic data in our study, such as age and genotype, demonstrate a random sample selection, which is similar to the patterns of other studies described in the literature, as in the study by Benzaken et al. (21), which was performed to estimate the current Brazilian population infected by HCV, or in European studies, such as the review by Messina et al. (22) and the cohort of Weidner et al. (23), presenting patients with a mean age of 58.4 ± 13.6 and the prevalence of genotype 1, 87.6% of all cases, and subtype 1B.

Hedenstierna et al. (24) described that among the main risk factors for persistence of advanced fibrosis after SVR was high BMI, as being overweight serves as an independent risk factor for hepatic steatosis in patients with HCC, as well as for increasing progression to fibrosis (25). In our cohort, the mean BMI was 26.6 ± 4.9 kg/m^2^, which is in line with data available in the literature (10, 23, 26, 27), and 57.9% of the patients were overweight. Ideal 19.3% obesity and 38.6% overweight; reduced number compared to data from other studies such as Do et al. (28), where of the more than 11,000 patients (96.2% men) beginning therapy, 78.0% of the patients were overweight (36.8% obese and 41.3% overweight), and in the study of Hu et al. (25), with 39.7% of patients overweight and 37.8% with obesity.

Hepatocytes are crucial for maintaining plasma glucose homeostasis; the main objective of our study was to investigate the impact of DAAs treatment and viral clearance on the metabolic profile in treated patients. Our findings in relation to the prevalence of dysglycemia in patients beginning therapy were high, when compared to most of the literature, being 43.8% pre-diabetic, against 12.5% diabetic, mainly due to stratification criteria; in one of the largest published reviews on the subject, with 62,354 patients, Ioannou et al. (27) found 22.0% of patients diagnosed with diabetes; similarly, in a meta-analysis carried out in 2018 by Ambachew et al. (29), involving 14,765 patients, the prevalence of DM2 among those infected with HCV was 19.6%. With a broader scope in the classification of patients, between pre-diabetes and diabetes, Yuan et al. (30) found findings of 25.5% for pre-diabetes and 8.16% for DM2; similarly, in the review by Weidner et al. (23), 27.0% of patients had changes in glycemic homeostasis at baseline, and obtained a reduction in fasting plasma glucose levels and HbA1c values independent of changes in BMI with therapy with DAAs. In the same vein, the study with stratification of patients based on the oral glucose tolerance test carried out by Huang et al. (31), with 683 patients, the prevalence of normoglycemia, altered fasting glucose, and DM2 was 27.7, 34.6, and 37.8%, respectively. HCV infection has a negative impact on glucose metabolism and increases IR, although the mechanism is unclear. Matsui et al. (32) demonstrated *in vitro* that hepatitis C viral replication negatively regulates hepatocyte nuclear factor 1α, suppressing GLUT2 expression and, therefore, reducing cellular glucose uptake.

Despite the non-significant reduction in related parameters of metabolic data (glycemia; HbA1c; HOMA-IR; insulin) before and after treatment (99.03 ± 21.87 vs. 96.39 ± 18.95; 5.50 ± 0.62 vs. 5.32 ± 0.98; 3.69 ± 3.28 vs. 3.56 ± 2.18; 15.18 ± 12.62 vs. 14.46 ± 8.52, respectively), our study is in agreement with most of the available studies, which showed an improvement in the glycemic profile in patients after HCV treatment (33–35); Hum et al. (36) observed in 2,435 patients that a reduction in HbA1c was significantly greater in the group that obtained SVR, as well as Ciancio et al. (10), who observed that despite the increase in weight, there was a significant reduction in the mean values of blood glucose and HbA1c, with 20.7% of patients being able to reduce or suspend their antidiabetic therapy, compared to the control group. In a subgroup of patients who had steatosis evaluation by elastography at both moments of the study, the blood glucose level was higher in patients with steatosis after treatment (*p* = 0.039). However, some authors did not observe an improvement in glycemic assessment (31, 37), such as Salomone et al. (38), in the follow-up of Italian patients, who did not observe variations in the GJ, but despite this, they reported an improvement in the IR profile; in the same way, Stine et al. (39) showed that HbA1c was not affected by the eradication of chronic HCV with DAAs in diabetic patients with and without cirrhosis. Long-term studies are necessary, as in an article recently published by Adinolfi et al. (40), with an average follow-up of 30 months, HCV elimination emerged as independently associated with a reduced risk of DM2. Another important point raised by Li et al. (41) would be the duration of the benefit in the long term, since in his study, in <3 years, there was a recovery in the level of HbA1c prior to SVR.

The direct link between HCV and the host's lipoproteins explains the significant interrelationship between HCV and the host's lipid metabolism. In our assessment, the significant increase in TC levels, mainly at the expense of LDL, is in accordance with clinical studies such as that of Meissner et al. (42), where a concomitant decrease in TGD and VLDL particle size and a marked increase in serum LDL have been reported after 24 weeks of treatment of patients infected with HCV genotype 1 treated with sofosbuvir (SOF)/RBV, regardless of the outcome treatment.

In the assessment of the lipid profile before and after treatment with IFN-PEG and RBV performed by Batsaikhan et al. (43), there was a difference between the group of patients with and without SVR. Patients who achieved SVR showed an average increase of 12.3 ± 51.6 mg/dl in the TGD level, but the group without SVR experienced an average increase of 1.2 ± 43.0 mg/dl. This may explain a direct effect of HCV clearance on lipid metabolism.

The evaluation time after starting therapy seems to be an important factor, since Mauss et al. (44) reported that the use of DAA regimens without IFN increased TC levels with no effect on triglycerides, while IFN-based therapy led to a reduction in TC levels with an increase in TGD, followed by an increase in levels of CT after reaching SVR.

Another relevant factor to be investigated are changes in the different lipid profile produced according to the type of DAA; when treating patients with HCV genotype 1, Hashimoto et al. (45) observed a rapid increase in LDL and CT during the first 28 days of treatment, which was stronger in patients who received LDV/SOF than in those who received daclatasvir/sunaprevir.

In assessing the degree of fibrosis using elastography, despite a high degree of hepatic stiffness (10.93 ± 11.70 kPa), the reduction found in our post-treatment study (8.94 ± 5.36 kPa with *p* = 0.006) is in agreement with other studies (46, 47). Although there is a short-term response, in the evaluation carried out by Kobayashi et al. (48), the degree of fibrosis was directly related to the follow-up time, with a progressive response at 12, 24, and 48 weeks after treatment (8.3, 7.4, and 5.3 kPa, respectively). Both the FIB-4 index and the APRI exhibited a strong correlation with the stage of hepatic fibrosis before FIB4 antiviral therapy (*r* = 0.319, *p* = 0.004; *r* = 0.287, *p* = 0.010; respectively), without maintaining the same correlation after therapy. Studies such as Hsu et al. (49) suggest that the rapid reductions observed in the values of APRI and FIB-4 may result mainly from the improvement of hepatic necroinflammation instead of fibrosis regression. The results of studies (20, 50) suggest that the non-invasive index values obtained after therapy with AADs may not be predictive of the concomitant stage of fibrosis, and an additional correlative study based on histology is needed to establish ideal cutoff points for predict the stages of fibrosis.

In the evaluation after obtaining SVR by transient elastography with CAP, the presence of significant steatosis was found in 66.7% of the patients and 23.1% had advanced fibrosis (F4), and our data are high when compared with the publication by Noureddin et al. (51), who reported that 47.5% of the 101 post-SVR patients had some degree of steatosis, of which 6.25% had advanced fibrosis. Another statistically significant finding in our results was the expected reduction in liver enzymes associated with SVR, similar to the work by Huynh et al. (52) where sustained normalization of ALT and AST was observed in 90.8% of patients at week 12 post-treatment. However, in a study with 834 patients, Welsch et al. (53) found that 35% of patients had continuous liver inflammation, determined by elevated levels of aminotransferase, despite HCV eradication.

The best form of segment for these patients who obtain SVR is still a topic that leaves a gap, due to the variability in the presentation of residual liver disease, both in the laboratory aspect and in imaging methods. In our study, the distribution of the degree of fibrosis among the patients evaluated before starting treatment was F0–F1 15.4%, F2 15.4%, F3 23.1%, and F4 46.2%, compared to a Brazilian study Castelo et al. (54), involving 313 monitored in the public health system, where 42.8% of patients who underwent TE had cirrhosis. In ratification of our findings, in the study conducted by Loo et al. (55) of the 411 patients evaluated at the beginning of treatment, 47.6% had basal fibrosis from F3 to F4. Current European and United States guidelines recommend only long-term follow-up in patients with the presence of advanced fibrosis in the pre-treatment and maintenance of elevated enzymes after treatment. In large centers, patients treated with DAA regimens can achieve SVR rates that approach 99%, being comparable to the results demonstrated in controlled clinical trials. However, according to Loo et al. (55), approximately two thirds of patients continue to need long-term follow-up due to advanced liver disease, despite viral eradication. These findings indicate the possibility of additional underlying chronic liver disease, for example, NAFLD or alcoholic liver disease, which can lead to further fibrosis progression and an increased risk of hepatocellular carcinoma.

Ongoing research and long-term follow-up studies are essential to determine the prognosis and management strategies of patients with chronic HCV infection after achieving treatment-induced viral eradication.

The most important limitations in this study are the sample size, which may not be able to reflect the real incidence of metabolic changes in patients with HCV, associated with the presence of basal changes in glycidic metabolism in the majority of individuals. On the other hand, there are further restrictions, like the absence of TE measures in all patients at the two assessment times and possible risk factor confusion, such as differentiation of analyses by genotype and the baseline disease treatment regimen.

The presence of a reduced and variable interval (12–52 weeks) for the evaluation after SVR is an important limiting factor, since the time difference between certain evaluations may not present early metabolic changes, with later neutrality, the opposite being true.

In conclusion, in this study, we observed that patients with hepatitis C already presented significant metabolic changes in the pre-treatment phase of the disease, a condition characterized mainly by the presence of changes in glucose and lipid metabolism present in about 50% of the sample. However, when assessing the impact that these changes could have in the post-treatment phase with direct antivirals, our data point to the importance, especially those related to the lipid profile.

Our results, although preliminary, reinforce the need both for screening the metabolic profile of these patients in the pre-treatment phase and for long-term follow-up, even when they evolve with a SVR, in most cases, with reduction of liver enzymes and fibrosis scores and degree. The permanence of metabolic glucose alterations and steatosis evaluated by the CAP in the post-treatment phase could negatively impact the disease progression and the evolution of these patients.

## Data Availability Statement

The original contributions presented in the study are included in the article/supplementary material, further inquiries can be directed to the corresponding author/s.

## Ethics Statement

The study was approved by the Research Ethics Committee of Federal University of Rio de Janeiro State.

## Author Contributions

PC: data collection and analysis. DG and CS: reading and statistical analysis. MC and CB-M: guidance and review. All authors contributed to the article and approved the submitted version.

## Conflict of Interest

The authors declare that the research was conducted in the absence of any commercial or financial relationships that could be construed as a potential conflict of interest.
